# Appraisal of Triglyceride-Related Markers as Early Predictors of Metabolic Outcomes in the PREVIEW Lifestyle Intervention: A Controlled *Post-hoc* Trial

**DOI:** 10.3389/fnut.2021.733697

**Published:** 2021-11-01

**Authors:** Santiago Navas-Carretero, Rodrigo San-Cristobal, Pia Siig Vestentoft, Jennie C. Brand-Miller, Elli Jalo, Margriet Westerterp-Plantenga, Elizabeth J. Simpson, Teodora Handjieva-Darlenska, Gareth Stratton, Maija Huttunen-Lenz, Tony Lam, Roslyn Muirhead, Sally Poppitt, Kirsi H. Pietiläinen, Tanja Adam, Moira A. Taylor, Svetoslav Handjiev, Melitta A. McNarry, Sylvia Hansen, Shannon Brodie, Marta P. Silvestre, Ian A. Macdonald, Nadka Boyadjieva, Kelly A. Mackintosh, Wolfgang Schlicht, Amy Liu, Thomas M. Larsen, Mikael Fogelholm, Anne Raben, J. Alfredo Martinez

**Affiliations:** ^1^Center for Nutrition Research, University of Navarra, Pamplona, Spain; ^2^Centro de Investigación Biomédica en Red (CIBER) obn, Instituto de Salud Carlos III, Madrid, Spain; ^3^IdiSNA, Navarra Institute for Health Research, Pamplona, Spain; ^4^Department of Nutrition, Exercise and Sports, Faculty of Science, University of Copenhagen, Copenhagen, Denmark; ^5^School of Life and Environmental Sciences and Charles Perkins Centre, The University of Sydney, Sydney, NSW, Australia; ^6^Department of Food and Nutrition, University of Helsinki, Helsinki, Finland; ^7^Department of Nutrition and Movement Sciences, NUTRIM, School of Nutrition and Translational Research in Metabolism, Maastricht University, Maastricht, Netherlands; ^8^Division of Physiology, Pharmacology and Neuroscience, MRC/ARUK Centre for Musculoskeletal Ageing Research, ARUK Centre for Sport, Exercise and Osteoarthritis, National Institute for Health Research (NIHR) Nottingham Biomedical Research Centre, School of Life Sciences, Queen's Medical Centre, Nottingham, United Kingdom; ^9^Department of Pharmacology and Toxicology, Medical University of Sofia, Sofia, Bulgaria; ^10^Applied Sports Technology, Exercise and Medicine (A-STEM) Research Centre, College of Engineering, Swansea, United Kingdom; ^11^Institute of Nursing Science, University of Education, Schwäbisch Gmünd, Germany; ^12^NetUnion Sarl, Lausanne, Switzerland; ^13^Human Nutrition Unit, Department of Medicine, School of Biological Sciences, University of Auckland, Auckland, New Zealand; ^14^Obesity Research Unit, Diabetes and Obesity Research Program, University of Helsinki and Endocrinology, Helsinki, Finland; ^15^Abdominal Center, Helsinki University Hospital and University of Helsinki, Helsinki, Finland; ^16^Cologne Center for Ethics, Rights, Economics, and Social Sciences of Health, University of Cologne, Cologne, Germany; ^17^Center for Health Technology Services Research (CINTESIS), NOVA Medical School, NMS, Universidade Nova de Lisboa, Lisboa, Portugal; ^18^Exercise and Health Sciences, University of Stuttgart, Nobelstraße, Germany; ^19^Steno Diabetes Centre Copenhagen, Gentofte, Denmark

**Keywords:** obesity, pre-diabetes, triglycerides (PubChem CID: 5460048), hypertriglyceridemic-waist phenotype, precision nutrition, diabetes, carbohydrate metabolism, lipid markers

## Abstract

**Background:** Individuals with pre-diabetes are commonly overweight and benefit from dietary and physical activity strategies aimed at decreasing body weight and hyperglycemia. Early insulin resistance can be estimated *via* the triglyceride glucose index {TyG = Ln [TG (mg/dl) × fasting plasma glucose (FPG) (mg/dl)/2]} and the hypertriglyceridemic-high waist phenotype (TyG-waist), based on TyG x waist circumference (WC) measurements. Both indices may be useful for implementing personalized metabolic management. In this secondary analysis of a randomized controlled trial (RCT), we aimed to determine whether the differences in baseline TyG values and TyG-waist phenotype predicted individual responses to type-2 diabetes (T2D) prevention programs.

**Methods:** The present *post-hoc* analyses were conducted within the Prevention of Diabetes through Lifestyle intervention and population studies in Europe and around the world (PREVIEW) study completers (*n* = 899), a multi-center RCT conducted in eight countries (NCT01777893). The study aimed to reduce the incidence of T2D in a population with pre-diabetes during a 3-year randomized intervention with two sequential phases. The first phase was a 2-month weight loss intervention to achieve ≥8% weight loss. The second phase was a 34-month weight loss maintenance intervention with two diets providing different amounts of protein and different glycemic indices, and two physical activity programs with different exercise intensities in a 2 x 2 factorial design. On investigation days, we assessed anthropometrics, glucose/lipid metabolism markers, and diet and exercise questionnaires under standardized procedures.

**Results:** Diabetes-related markers improved during all four lifestyle interventions. Higher baseline TyG index (*p* < 0.001) was associated with greater reductions in body weight, fasting glucose, and triglyceride (TG), while a high TyG-waist phenotype predicted better TG responses, particularly in those randomized to physical activity (PA) of moderate intensity.

**Conclusions:** Two novel indices of insulin resistance (TyG and TyG-waist) may allow for a more personalized approach to avoiding progression to T2D.

**Clinical Trial Registration:** https://clinicaltrials.gov/ct2/show/NCT01777893 reference, identifier: NCT01777893.

## Introduction

Pre-diabetes and type 2 diabetes (T2D) with accompanying metabolic complications, such as hyperglycemia, insulin resistance, disturbances in lipid metabolism, and pro-inflammatory processes, are associated with the obesity pandemic (1, 2). Pre-diabetes is defined as impaired fasting glucose and/or glucose intolerance or/and elevated glycosylated hemoglobin (HbA1c) with or without overweight or obesity. More targeted preventive and management interventions require investigation with the aim of improved metabolic outcomes (3). Individuals with obesity, pre-diabetes, and T2D benefit from lifestyle interventions involving dietary and physical activity (PA) programs, in which macronutrient distribution, glycemic index (GI), and fiber consumption are prescribed, within energy-restricted diets (4–6). In addition, various PA programs incorporating different exercise types are also beneficial (7, 8).

Indeed, clinical and epidemiological evidence highlights independent or combined beneficial effects of manipulating dietary patterns (quantity and composition of the nutritional intake) and/or PA (type, intensity, and/or frequency) to improve glucose homeostasis and reduce diabetes incidence in people with pre-diabetes (9–11). Specifically, previous lifestyle interventions, such as the Chinese Da Qing study, the Finnish Diabetes Prevention Study, and the US, Indian, and Japanese programs have demonstrated evidence in precluding and delaying progression to T2D and associated morbidities (12). Furthermore, a lifestyle approach was shown to reduce all-cause mortality after 30 years of follow-up (13).

Clinical trials implementing tailored dietary and PA prescriptions for T2D prevention and management have shown inter-individual differences depending on the pre-diabetes grade and associated phenotypes (3). Differences in pretreatment fasting glucose and insulin status have been reported to be associated with weight loss success in patients consuming low GI or fat-rich diets (14). Additionally, data-driven cluster analysis using variables related to glucose utilization identified five subgroups within adult-onset diabetes populations providing some potential for precision nutritional management based on personalized phenotypes (15).

A scenario of tailored treatment also requires the availability of simple, reliable, and inexpensive tests to detect insulin resistance. A novel index based on glucose and TG measurements (the TyG index), has shown high sensitivity and specificity for identifying participants with disturbed insulin function (16), as well as early diabetes onset in patients with normal fasting glucose (17). Waist circumference (WC) has also emerged as a useful clinical marker to distinguish patients according to cardiometabolic risks (18). A phenotype characterized by both high WC and high TyG index has been associated consistently with abnormal glucose and insulin metabolism, with a potential for timely detection of diabetes and more personalized interventions (19).

The PREVIEW trial (20) explored whether a nutritional intervention based on different protein intakes and dietary GI in combination with PA at different intensities, after an initial weight loss produced long-term benefits for T2D prevention. All participants had pre-diabetes and were overweight or obese at baseline. While the incidence of T2D after 3 years of intervention was very low in all four-intervention groups, reversion to normal glucose status varied significantly between the two diet groups (21). In the current *post-hoc* analysis, the TyG index and the high WC high TyG phenotype were tested as a potential predictor of clinical responses concerning lifestyle modification at the end (3 years) of the PREVIEW intervention. In addition, we aimed to specifically identify inter-individual differences in order to personalize lifestyle prescriptions with a focus on precision nutrition. We hypothesized that different intervention patterns may have differential effects depending on the baseline phenotype.

## Materials/Subjects and Methods

The present *post-hoc* analyses were conducted within the PREVIEW study (NCT01777893) which has been described elsewhere (20). In summary, this study was a multi-center randomized trial conducted at eight sites in eight different countries, aiming to reduce the incidence of T2D in a population with pre-diabetes (21). The 3-year randomized intervention trial was implemented in two phases. First, a 2-month weight loss phase using a commercial low-energy diet (LED) (~3.4 MJ ≪800 kcal≫ per day) to achieve an ≥ 8% bodyweight reduction (22). Second, a 34-month intervention for weight loss maintenance encompassing four intervention arms with two diets and two PA programs using a 2 x 2 factorial design (21). Given the nature of the study, and the final outcome, it was decided that no control group would be needed, and benefits of participating in the intervention were expected in the four intervention arms (20).

The sample size was calculated to be 2,403 subjects, taking into account data from the Finnish Diabetes Prevention Study (DPS) and US Diabetes Prevention Program (DPP), and anticipating a 3-year incidence of T2D in our population of 21%, as described in detail in the Methods article of the PREVIEW Study (20). Once the potential participants were confirmed eligible, they were enrolled in the trial and randomized to one of the four treatment groups. Randomization was stratified by gender and age group (25–54, and 55–70 years of age), and sequentially assigned from each stratum to different interventions, hence, securing an even distribution of gender and age group over the four intervention arms in each center (20).

A total of 2,326 participants were enrolled in the intervention; of those 2,223 started the trial, and completed CID7 899. These participants were adults (25–70 years), overweight [body mass index (BMI) ≥ 25 kg/m^2^] men and women with pre-diabetes as defined by the American Diabetes Association (ADA): fasting plasma glucose (FPG) of 5.6-−6.9 mmol/L and/or of 7.8-−11.0 mmol/L 2 h after an oral glucose tolerance test (OGTT) of 75 g glucose when FPG <7.0 mmol/L (23).

Clinical investigation days (CID) were conducted in the facilities of the intervention centers at baseline (CID1), 2 months (CID2, end of the first phase), 6 months (CID3), 12 months (CID4), 18 months (CID5), 24 months (CID6), and 36 months (CID7, end of the trial). In the present study, only data from the first and the last visits were used in the analyses. At each CID, anthropometry, blood samples, 24-h urine samples, 7-days accelerometer data, dietary records, and questionnaires were collected. In addition, participants attended 17 group visits led by trained instructors who supported the acquisition of new diet and physical activity (PA) habits. A behavior modification tool (PREMIT) was developed specifically for PREVIEW (24). All centers had approval from their respective Research Ethics Boards before starting the trial, and all participants were required to give written informed consent in their mother tongue before enrollment (20). The study was conducted in accordance with the ethical standards of the appropriate ethics committee of each center, and following the Helsinki Declaration of 1975 as revised in 1983.

Those participants who reached ≥8% body weight loss during the low-energy diet (LED) phase (*n* = 1,856), proceeded with the 34-month weight maintenance phase in one of the four intervention arms to which they were randomly allocated at the beginning of the study. The two diets were consumed *ad libitum* having received guidance on the proportion of foods to consume from different food categories in order to achieve the following nutritional composition: (1) Moderate protein (MP) diet (15% of energy – E% - from protein, 30 E% fat, 55 E% carbohydrates, and moderate GI, (2) High protein (HP) diet (25 E% protein, 30 E% fat, 45 E% carbohydrates, and lower GI). The two PA groups, both with a comparable energy expenditure of >4,200 kJ/week, were as follows: high-intensity (HI) PA (75 min/week) and moderate-intensity (MI) PA (150 min/week).

### Data Collection and Processing

All measures and samples were collected at the intervention centers during the CIDs following Standard Operation Procedures (SOP) specifically designed for the study (20). Blood samples were collected from the antecubital vein after fasting for 10–12 h. At baseline, month 6, and years 1, 2, and 3, an oral glucose tolerance test (OGTT) with 75 g glucose was performed, with blood sample collection at every 30 min over the subsequent 2 h, although in the present analysis, only data from baseline and 3 years are analyzed. Blood samples were initially stored at −80°C and then sent to the central laboratory of the project in Finland (National Institute for Health and Welfare, Helsinki) for the analysis of FPG, glycosylated hemoglobin (HbA1c), insulin, homeostatic model assessment of insulin resistance (HOMA-IR), lipids (total, HDL and LDL cholesterol, triglycerides -TG-), and C-reactive protein, as previously described (20). Insulin was measured in Denmark (University of Copenhagen) on Siemens Immulite 2000 equipment, Siemens Healthcare, Diagnostic products, Gwynedds, UK following the Immuno-Chemiluminescent method as previously described (21).

During the CIDs, anthropometric and blood pressure measurements were also collected (22). Standardized measures for body weight, body composition (fat mass and fat-free mass by Bioelectrical Impedance Analysis and DEXA, height, waist, and hip and thigh circumference were performed in light clothes by trained researchers, following the guidance described in the specific SOP developed for PREVIEW. Systolic blood pressure (SBP) and diastolic blood pressure (DBP) were measured on the right arm with a validated automatic device after 5–10 min in a resting position (21). The details concerning the analyses estimating reported energy intake *via* 4-day food records and actual protein consumption, accelerometer outcomes, and those explanations involving the Baecke questionnaire implementation have been described elsewhere (21).

The triglyceride glucose index (TyG) and the TyG-waist circumference index (TyG-waist) were estimated as surrogate markers of insulin resistance reflecting the individual physiological status (25). The TyG and TyG-Waist were calculated using the following formulas: TyG = Ln [TG (mg/dl) × FPG (mg/dl)/2] (16) and TyG-Waist = TyG index × WC (cm) (26).

### Statistical Methods

Baseline descriptive variables of completers are shown as mean ± SD, with ANOVA used to assess the effect of age and sex (Table 1). To determine the influence of the intervention within each arm (diet and PA groups), a 2 x 2 factorial ANOVA was performed on anthropometrical and biochemical measures for participants who provided data at baseline and CID7 (3 years). Linear regression models were fitted to changes in anthropometrical and biochemical measures from baseline to the end of the study. In the first model, we adjusted for age, sex, and baseline values of the dependent variable. Intervention center and change in body mass index (BMI) were added as covariates in Model 2. These regression analyses also considered the interaction between diet (energy and protein intake) and PA (Baecke questionnaires).

**Table 1:** Baseline characteristics of PREVIEW completer participants categorized by age and sex.

	**Total (*N* = 899)**	**Female**		**Male**		***p*-value for sex^**a**^**	***p*-value for age^**a**^**	***p*-value for int.^**b**^**
		**<55 years** **(*N* = 263)**	**>55 years** **(*N* = 309)**	**<55 years** **(*N* = 127)**	**>55 years** **(*N* = 200)**			
BMI (kg/m^2^)	33.4 (5.1)	34.4 (5.7)	32.9 (4.8)	33.8 (5.0)	32.4 (4.3)	0.053	**<0.001**	0.838
Waist circumference (cm)	108.0 (12.8)	104.2 (12.9)	105.3 (11.7)	113.3 (12.6)	113.8 (11.1)	**<0.001**	0.258	0.683
Waist/hip ratio	0.94 (0.09)	0.88 (0.07)	0.91 (0.07)	1.00 (0.07)	1.03 (0.06)	**<0.001**	**<0.001**	0.777
FPG (mg/ml)	111.1 (11.8)	106.3 (11.6)	112.3 (10.5)	112.7 (14.4)	114.6 (10.2)	**<0.001**	**<0.001**	**0.011**
2h glucose (mg/ml)	136.1 (38.3)	130.4 (35.0)	139.9 (39.2)	135.3 (39.1)	138.2 (40.0)	0.576	**0.005**	0.219
Insulin (mIU/L)	12.3 (7.2)	12.0 (6.8)	11.2 (5.7)	14.8 (11.0)	12.6 (6.4)	**<0.001**	**0.007**	0.134
HOMA-IR	3.41 (2.24)	3.19 (1.94)	3.15 (1.77)	4.24 (3.71)	3.58 (1.86)	**<0.001**	0.078	**0.048**
HOMA-beta	11.3 (6.3)	11.6 (6.4)	10.3 (5.1)	13.4 (8.8)	11.3 (5.7)	**0.005**	**<0.001**	0.368
TG (mg/dl)	127 (61)	116 (59)	129 (56)	146 (76)	128 (58)	**0.004**	0.62	**<0.001**
Total cholesterol (mg/dl)	200 (39)	196 (34)	211 (41)	195 (40)	193 (38)	**<0.001**	**0.001**	**0.001**
TyG index	8.76 (0.47)	8.61 (0.49)	8.80 (0.42)	8.89 (0.52)	8.81 (0.43)	**<0.001**	**0.002**	**<0.001**
TyG-waist index	947.3 (132.0)	898.0 (130.1)	928.0 (118.8)	1008.2 (132.6)	1003.0 (118.6)	**<0.001**	**0.036**	**0.045**
Baecke index	7.29 (1.46)	7.15 (1.52)	7.46 (1.47)	7.19 (1.39)	7.34 (1.41)	0.814	**0.015**	0.469
Protein intake (g/d)	92.0 (27.4)	88.5 (23.7)	83.5 (19.9)	109.5 (39.0)	98.9 (26.8)	**<0.001**	**<0.001**	0.137
EIR (kJ/d)	8,690 (2,351)	8,744 (2,326)	7,836 (1,924)	10,003 (2,924)	9,121 (2,075)	**<0.001**	**<0.001**	0.941

*P-values show significant differences and interactions*.

a *ANOVA analysis*.

b *p-value for interaction*.

*BMI, Body Mass Index; FPG, Fasting Plasma Glucose; TG, Triglycerides; EIR, Energy intake reported. Bold values mean that p-value is significant (< 0.05)*.

Participants were subsequently categorized into two groups according to the median values of the TyG and TyG-waist index (8.8 for TyG and 943.6 for TyG-waist, respectively) in order to assess independently the predictive value of these trygliceride-related markers and the interaction with the intervention arms. Linear regression analyses for the difference between baseline and endpoint were carried out to assess the estimated marginal means for the anthropometric and metabolic markers. The regression model included the interaction term of the two TyG categories and each one of the intervention arms (in separate models) adjusted for age, sex, baseline BMI, and intervention center.

The results of per protocol analysis were preferred because they better reflect the effects of the intervention when considered optimally, decreasing the probability of incurring a type II error, as described elsewhere (27, 28). The statistical per protocol analyses and calculations were carried out using the statistical program, R version 4.0.2 (R Core Team, Vienna, Austria) using the following packages “dplyr,” “psych,” “DescTools,” “tidyr,” “ggplot2,” “ggpubr,” “MASS,” “multcomp,” “emmeans,” and RStudio version 1.2.1335. The *P* < 0.05 was considered statistically significant.

## Results

Baseline characteristics of the completers (*n* = 899) in the analysis are shown in Table 1. Sex, age, body mass index (BMI), and WC correlated as expected with specific metabolic markers of glucose homeostasis and insulin resistance. Energy intake and physical activity (PA) (assessed by Baecke estimation) were also significant predictors. The TyG (*p* < 0.001) and the TyG-waist index (*p* = 0.045) showed significant interactions with age and sex with a modification of the effects mediated by both factors (Table 1).

Changes in adiposity, glucose, and TG markers in the intervention arms from baseline to the end of the study are shown in Table 2. There were no differences in changes in glucose, BMI, and WC among the four interventions groups (Table 2). However, there were greater reductions in TG (*P* = 0.004), TyG (*p* = 0.023), and TyG-waist (*p* = 0.061) in the moderate PA group. The Baecke scores showed that protein intake and energy intake varied as expected according to the intervention group (Table 2).

**Table 2:** Change in anthropometrical and biochemical variables during the PREVIEW intervention between baseline and after 36-months of intervention (CID7), categorized by intervention arm.

**Metabolic variables**	**Intervention arms**					
	**Protein intake**			**Physical activity intensity**		
	**Moderate (*n* = 452)**	**High (*n* = 447)**	***p* for protein^**a**^**	**Moderate (*n* = 448)**	**High (*n* = 451)**	***p* for intensity^**a**^**
Δ BMI (kg/m^2^)	−1.64 (2.44)	−1.72 (2.62)	0.648	−1.75 (2.58)	−1.61 (2.48)	0.42
Δ Waist circumference (cm)	−3.90 (7.43)	−3.42 (8.44)	0.371	−3.99 (7.86)	−3.33 (8.03)	0.21
Δ FPG (mg/ml)	−1.55 (12.92)	−1.40 (11.63)	0.855	−1.95 (12.21)	−1.01 (12.37)	0.25
Δ TG (mg/dl)	−17.35 (54.70)	−12.07 (49.15)	0.129	−19.72 (56.95)	−9.76 (46.21)	**0.004**
Δ TyG	−0.15 (0.42)	−0.13 (0.39)	0.379	−0.17 (0.44)	−0.11 (0.38)	**0.023**
Δ TyG-waist	−48.88 (91.12)	−42.26 (96.84)	0.292	−51.47 (96.05)	−39.74 (91.68)	0.061
Δ Baecke index	0.61 (1.15)	0.52 (1.18)	0.365	0.58 (1.05)	0.54 (1.28)	0.682
Δ Protein intake (g/day)	−7.74 (26.36)	−14.67 (28.36)	**0.001**	−10.25 (26.85)	−12.13 (28.27)	0.347
Δ EIR (kJ/day)	−1886 (2172)	−1888 (2319)	0.987	−1763 (2273)	−2009 (2214)	0.129

a *ANOVA 2 × 2*.

*BMI, Body Mass Index; FPG, Fasting Plasma Glucose; TG, Triglycerides; EIR, Energy Intake reported. Bold values mean that p-value is significant (< 0.05)*.

The regression models when adjusted by age, sex, baseline value of the dependent variable, intervention center, and BMI changes did not demonstrate any favorable effects of a high protein diet compared with a moderate protein diet on changes in fasting glucose (*p* = 0.242) or circulating TG (*p* = 0.181) concentrations (Table 3).

**Table 3:** Regression models concerning the effect of intervention arms (diet and PA groups) on the variation of anthropometrical and biochemical markers as dependent variables after 36 months of intervention.

	**Protein intake arms**			**Physical intensity arms**			***p* for interaction**
	**β**	**SE**	***P***	**β**	**SE**	***p***	
**Δ** **Body Mass Index (kg/m**^**2**^**)**							
Model 1	−0.027	0.237	0.910	0.185	0.235	0.432	0.728
Model 2	−0.073	0.232	0.752	0.166	0.231	0.472	0.704
**Δ** **Waist circumference (cm)**							
Model 1	0.757	0.733	0.302	0.908	0.729	0.213	0.648
Model 2	0.673	0.489	0.169	0.441	0.486	0.365	0.951
**Δ** **FPG (mg/ml)**							
Model 1	0.816	1.022	0.425	1.711	1.019	0.093	0.313
Model 2	1.106	0.945	0.242	1.484	0.941	0.115	0.275
**Δ** **Triglycerides (mg/ml)**							
Model 1	4.429	4.124	0.283	7.614	4.110	0.064	0.751
Model 2	5.035	3.758	0.181	6.516	3.743	0.082	0.606
**Δ** **TyG index**							
Model 1	0.023	0.035	0.516	0.058	0.035	0.096	0.808
Model 2	0.030	0.030	0.318	0.046	0.030	0.123	0.666
**Δ** **TyG-waist index**							
Model 1	10.168	8.613	0.238	14.334	8.574	0.095	0.674
Model 2	10.260	5.689	0.072	9.097	5.658	0.108	0.964

*Model 1: adjusted by age, sex, and baseline value of dependent variable; Model 2: adjusted by age, sex, baseline value of dependent variable, intervention center, and ΔBMI (between baseline and month 36); FPG: Fasting Plasma Glucose*.

**Figure 1:**
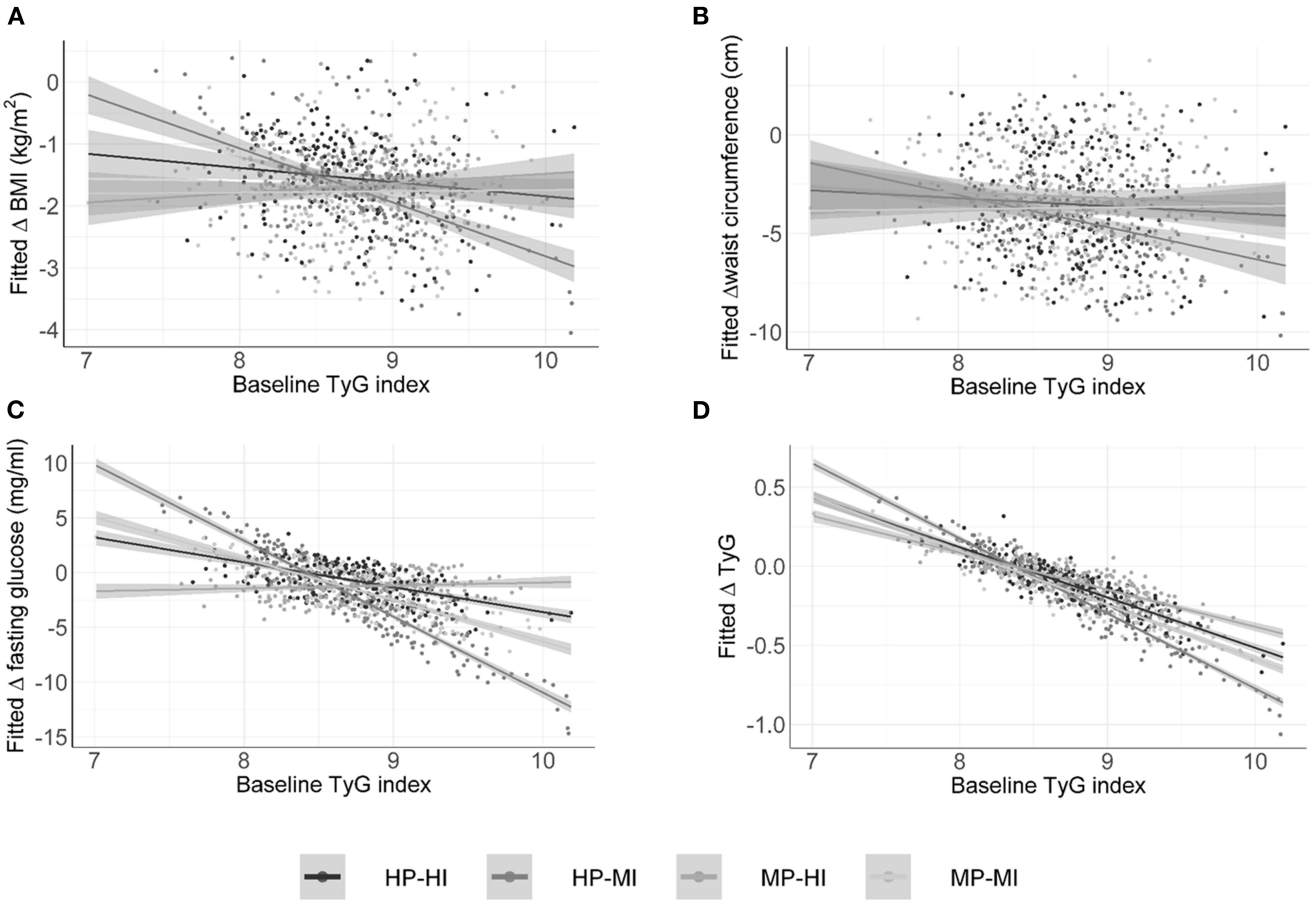
Baseline triglyceride glucose index (TyG) prediction value for changes in anthropometric and metabolic outcomes categorized by the intervention groups of the PREVIEW study. HP, high protein; MP, moderate protein; HI, high intensity; MI, moderate intensity. **(A)** Fitted change in BMI; **(B)** Fitted change in waist circumference; **(C)** Fitted change in fasting glucose; **(D)** Fitted change in triglycerides.

**Figure 2:**
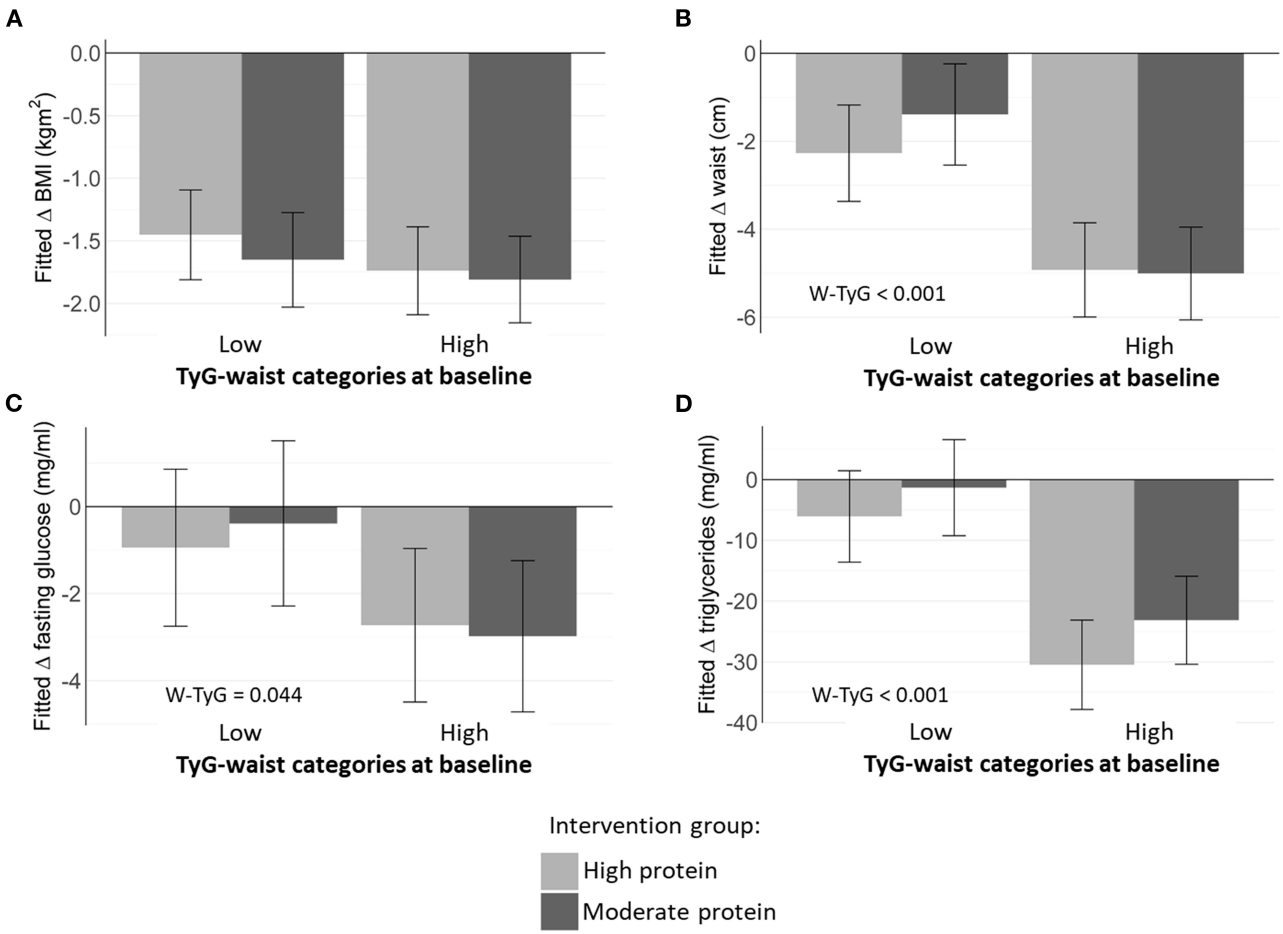
Estimated marginal means of changes in metabolic markers depending on baseline TyG-waist categories and dietary protein intake (high vs. moderate intervention group). **(A)** Fitted change in BMI; **(B)** Fitted change in waist circumference; **(C)** Fitted change in fasting glucose; **(D)** Fitted change in triglycerides.

**Figure 3:**
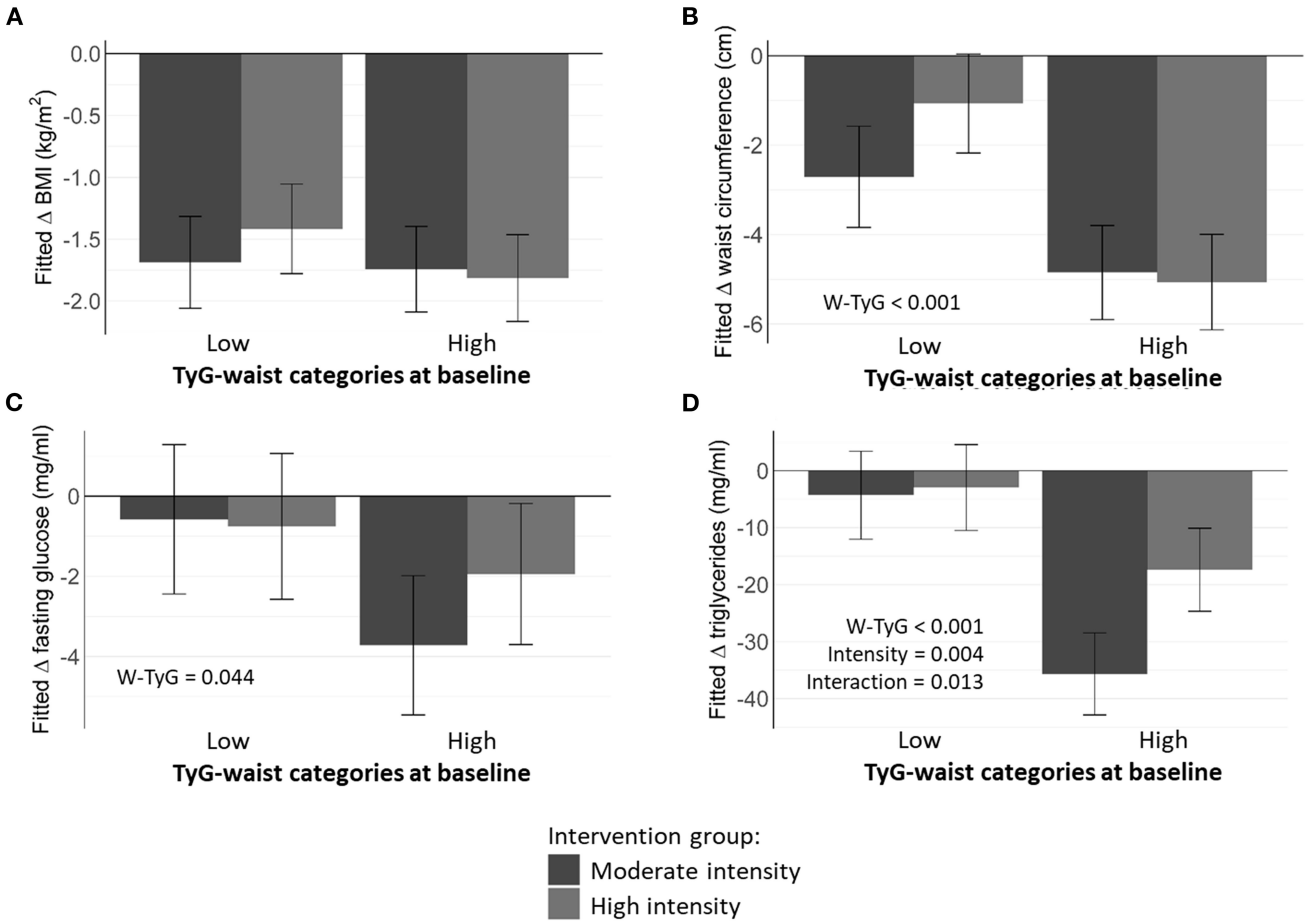
Estimated marginal means of metabolic markers depending on baseline TyG- waist categories and physical activity (high vs. moderate). **(A)** Fitted change in BMI; **(B)** Fitted change in waist circumference; **(C)** Fitted change in fasting glucose; **(D)** Fitted change in triglycerides.

Elevated TyG index at baseline independently and significantly predicted greater reductions in BMI, FPG, and TG, but not WC, within each intervention group (Figure 1). Furthermore, a higher baseline TyG-waist phenotype was associated with greater decreases in WC, FPG, and circulating TG, irrespective of the protein level (Figure 2). Moderate intensity PA (Figure 3) predicted more favorable metabolic effects than more intensive PA. Remarkably, those participants with higher baseline TyG-waist values responded better overall to all lifestyle interventions (Figures 2, 3).

## Discussion

Individuals with pre-diabetes and higher baseline TyG and TyG-waist phenotypes had superior outcomes in all four intervention groups in this secondary analysis of the PREVIEW Study. An elevated TyG index at baseline independently predicted greater reductions in body mass index (BMI), FPG, and TG, but not WC, within all four intervention groups. Furthermore, a higher baseline TyG-waist phenotype was associated with greater decreases in WC, FPG, and circulating TG, irrespective of the protein intake. Moderate physical activity (PA) intensity also appeared to be more efficacious than high intensity (HI) PA intervention arms with respect to improvements in TG and WC. However, we did not find any association between the baseline phenotypes (TyG index or TyG-waist phenotype) and response to different treatments.

Obesity and accompanying inflammatory phenomena are associated with hyperglycemia and insulin resistance, providing early manifestations that often lead to type-2 diabetes (T2D) and cardiovascular events (29, 30). Evidence suggests that there are also differences in prognosis associated with diabetes prevention and treatment that are due to inter-individual heterogeneity in glucose tolerance (3). We hypothesized that novel markers of insulin resistance, the TyG index and the TyG-waist phenotype, may help to define and individually categorize patients. Trait differences among patients may appear depending on personal (epi)genotype, microbiota, and phenotypic characteristics. A better understanding of these effects may contribute to tailoring more effective therapeutic interventions for each individual (31). Indeed, the growing emphasis on precision nutrition is based on the premise that clinical variability may require personalized management (32).

Individualized approaches could be supported by “omics” technologies (33, 34) and by clustering patients according to pretreatment weight-for-height, WC, homeostasis model assessment (HOMA), TyG, and other phenotype-based algorithms (15, 35, 36). Recently, a machine-learning approach integrating anthropometrics, blood biochemistry, diet, PA, and gut microbiota composition accurately forecasted personalized post-prandial glycemic response to a meal (37). Precision medicine methodologies may also demonstrate that similar dietary prescriptions cause different weight loss outcomes depending on pre-treatment FPG levels (38), while the precision of clinical monitoring of glucose tolerance and glycosylated hemoglobin (HbA1c) may be useful for pre-diabetes management (39).

The implementation of supervised lifestyle interventions in individuals with pre-diabetes usually focuses on weight loss (5–10% of body mass) by prescribing energy-controlled dietary regimes and increases in exercise expenditure in order to reduce blood glucose levels (40). Less is known about the prescription of dietary patterns with different macronutrient distribution, glycemic loads, or restriction of specific foods. Similarly, we are yet to learn whether the different forms of PA, including moderate vs. vigorous intensity, resistance training, or other health behaviors will differentially improve markers of the metabolic syndrome, including diabetes (41, 42).

Diabetes risk scores and diagnostic algorithms for personalized precision medicine often use factors, such as age, sex, family clinical history, WC, BMI, smoking, and alcohol consumption, dietary and PA habits, as well as blood pressure, lipid and glucose markers (3, 43). Distinctive pathophysiological profiles in pre-diabetes phenotypes have been described based on insulin secretion, insulin sensitivity, and beta-cell dysfunction assessed by IFG, IGT, and Hb1Ac measurements (40). More recently, HOMA, TyG, and TyG-waist phenotypes have been considered with the goal of categorizing different degrees of insulin resistance and glucose status (17) across different patients. Circulating fatty acids and TG have been associated with insulin sensitivity and glucose tolerance, *via* the generation of toxic lipids and inflammatory molecules. Changes in cellular membranes have also been implicated through alterations in glucose transport and the insulin signaling cascade, endoplasmic reticulum stress, and reactive oxygen species (44). The glucose-fatty acid cycle may also be disturbed *via* circulating lipid species and inflammatory mediators (45).

Most randomized trials have focused on weight lowering under energy restriction or dietary recommendations concerning the nutritional quality of the consumed foods with an emphasis on fat and carbohydrate/fiber intake as well as general non-personalized instructions for PA of different types and intensities (12). However, less scientific evidence is available on the role of protein intake and its interactions with the level of PA after weight loss is achieved.

The impact of higher protein intake on TG (a marker of lipid metabolism) and the TyG index (as an affordable surrogate of insulin resistance) requires further study. Previous clinical and lifestyle intervention trials have combined multidisciplinary prescriptions, including dietary modifications, with or without energy restriction, with approaches to enhance daily PA or exercise training, making it difficult to separately discriminate the role of each factor (diet vs. PA). These difficulties were overcome in the PREVIEW project (21), where four experimental arms were designed: two levels of protein consumption and two intensities of PA after following a low energy diet (LED) to induce at least 8% weight loss in 2 months (22). Regarding protein intake, the main limitation encountered was that the group following a high protein intake pattern reduced their protein consumption toward the end of the study, which makes it difficult to interpret the results. These findings build on previous studies that suggest replacing sitting with low intensity PA to produce metabolic benefits that contribute to the prevention and management of T2D (46). However, despite differences in instruction [moderate intensity (MI) vs. high intensity (HI) PA], there were no actual differences in PA intensity. Furthermore, total PA was as strongly associated with cardiometabolic risk markers as moderate- to vigorous-intensity physical activity (MVPA), which implies that the accumulation of total PA over the day is more important than the intensity of PA in this population (47).

Secondary analyses in the Look AHEAD and PREDIMED studies, which were focused on reducing cardiometabolic events by means of lifestyle interventions revealed improvements in circulating glucose and TG even after corrections for the degree of weight change (48, 49). And vice versa, most of the previous trials revealed that weight loss seems to be an independent predictor for the prevention of type 2 diabetes mellitus (T2DM), even after adjustments for diet and PA. The PREVIEW project showed that after a period of weight loss, there were no separate effects of diet composition or PA intensity on the risk of developing T2D within 3 years. An unexpected finding in PREVIEW was that significantly fewer participants achieved normoglycemia in the high protein high intensity (HPHI) PA group, compared to the other three groups. However, a limitation of the PREVIEW study is the high attrition rate and the possibility of selection bias (e.g., in the recruitment of more health-conscious participants). Nonetheless, the results appear robust and plausible, providing new evidence that clinicians should consider in a personalized fashion.

Several clinical and epidemiological studies have reported that the TyG index was superior to FPG and TG alone in predicting metabolically unhealthy individuals with fasting glucose levels under 5.5 mmol/L (16, 17, 36), as well as a role for TyG-waist for categorizing the hypertriglyceride phenotype (18, 19). Our findings suggest that the intensity of PA has a role in the management of glucose and lipid status. Furthermore, the conceptual framework underlying precision medicine is founded on assessing inter-individual heterogeneity (50). Accounting for phenotypic variability requires an understanding of the markers to be considered (51) and the characteristics of the intervention (52). In summary, both TyG indices count with sufficient scientific evidence as markers of insulin resistance, which showed similar or better precision than FPG, TG, or WC alone (16, 53). Indeed, these indices are as reliable as homeostatic model assessment for insulin resistance (HOMA-IR) regarding insulin resistance, but additionally are simpler, easier to interpret, and inexpensive than analyzing insulin or HOMA, as well as more informative than glucose measurements (16, 54).

In conclusion, individuals with pre-diabetes and higher baseline TyG and TyG-waist had better outcomes in all four PREVIEW intervention arms. This is in agreement with the commonly accepted clinical practice that patients with extreme features of the metabolic syndrome respond earlier and better to diverse dietary or pharmacological treatments. In contrast, we did not find any association between the baseline phenotype (TyG-index of TyG-waist) and response to different treatments. A goal of this research was to prospectively analyze the value of the baseline insulin resistance status based on both serviceable TyG tools to characterize patients at baseline and explain future outcomes, in order to facilitate a personalized prescription for application in subjects with pre-diabetes based on glucose tolerance status.

## Data Availability Statement

The raw data supporting the conclusions of this article will be made available by the authors, without undue reservation.

## Ethics Statement

The studies involving human participants were reviewed and approved by Denmark: the Research Ethics Committees of the Capital Region; Finland: Coordinating Ethical Committee of HUS (Helsinki and Uusimaa Hospital District); the UK: UK National Research Ethics Service (NRES) and East Midlands (Leicester) Ethics Committee; Netherlands: Medical Ethics Committee of the Maastricht University Medical Centre; Spain: Research Ethics Committee of the University of Navarra; Bulgaria: Commission on Ethics in Scientific Research with the Medical University-Sofia (KENIMUS); Australia: The University of Sydney, Human Research Ethics Committee (HREC); and New Zealand: Health and Disability Ethics Committees (HDEC). The patients/participants provided their written informed consent to participate in this study.

## Author Contributions

AR, JM, MF, JB-M, MW-P, IM, NB, SH, GS, WS, and SP participated in the study design. Specifically, AR and JM were coordinators and led the objectives of this manuscript. SN-C, RS-C, PS, EJ, LS, TH-D, MH-L, TL, RM, KP, TA, MT, MM, SH, SB, MS, KM, AL, and TML conducted the study and provided data necessary for the study. JM and RS-C conducted the statistical analyses. SN-C, RS-C, and JM performed the preliminary writing of the manuscript. All authors read and approved the final version of the manuscript.

## Funding

This work was supported by the EU framework program 7 (FP7/2007-2013) grant agreement # 312057, National Health and Medical Research Council - EU Collaborative Grant, AUS 8, ID 1067711), the Glycemic Index Foundation Australia through royalties to the University of Sydney, the New Zealand Health Research Council (14/191) and University of Auckland Faculty Research Development Fund, the Cambridge Weight Plan© donated all products for the 8-weeks LED period, the Danish Agriculture & Food Council, the Danish Meat and Research Institute, National Institute for Health Research Biomedical Research Centre (NIHR BRC) (UK), Biotechnology and Biological Sciences Research Council (BBSRC) (UK), Engineering and Physical Sciences Research Council (EPSRC) (UK), Nutritics (Dublin) donated all dietary analyses software used by UNOTT. Juho VainioFoundation (FIN), Academy of Finland (Grant Numbers: 272376, 314383, 266286, 314135), Finnish Medical Foundation, Gyllenberg Foundation, Novo Nordisk Foundation, Finnish Diabetes Research Foundation, University of Helsinki, Government Research Funds for Helsinki University Hospital (FIN), Jenny and Antti Wihuri Foundation (FIN), Emil Aaltonen Foundation (FIN). CIBEROBN of Spain is also gratefully acknowledged. RS-C acknowledges financial support from the Juan de la Cierva Program Training Grants of the Spanish State Research Agency of the Spanish Ministerio de Ciencia e Innovación y Ministerio de Universidades (FJC2018-038168- I). The funders of the study had no role in the study design, data collection, data analysis, data interpretation, or writing of the report.

## Conflict of Interest

TL was employed by company NetUnion Sarl. PS has received travel grants from the Cambridge Weight Plan, UK. JB-M is the President and Director of the Glycemic Index Foundation, oversees a glycemic index GI testing service at the University of Sydney and is a co-author of books about diet and diabetes. IM is a member of the UK Government Scientific Advisory Committee on Nutrition, Treasurer of the Federation of European Nutrition Societies, Treasurer of the World Obesity Federation, member of the Mars Scientific Advisory Council, member of the Mars Europe Nutrition Advisory Board, and Scientific Adviser to the Waltham Centre for Pet Nutrition. He is also a member of the Nestle Research Scientific Advisory Board and of the Novozymes Scientific Advisory Board. SP was the Fonterra Chair in Human Nutrition and Principle Investigator for NZ National Science Challenge High Value Nutrition during the PREVIEW intervention. TML is an advisor for the “Sense” diet program. AR has received honorariums from Novo Nordisk A/S, the International Sweeteners Association, Nordic Sugar and Unilever. JM is the President of IUNS. The remaining authors declare that the research was conducted in the absence of any commercial or financial relationships that could be construed as a potential conflict of interest.

## Publisher's Note

All claims expressed in this article are solely those of the authors and do not necessarily represent those of their affiliated organizations, or those of the publisher, the editors and the reviewers. Any product that may be evaluated in this article, or claim that may be made by its manufacturer, is not guaranteed or endorsed by the publisher.

## Supplementary Material

Supplementary Table 1 CONSORT Statement checklist, as required by the ICMJE.

Supplementary Figure 1 Flowchart of the volunteers involved in the study, and whose data were analysed in the present article (Screened, Eligible and Completers, as well as those with valid full data).

## Abbreviations

ADA: American Diabetes Association
BMI: body mass index
CID: Clinical Investigation Day
DP: diabetes prevention
EIR: energy intake reported
FPG: fasting plasma glucose
GI: glycemic index
HbA1c: glycosylated hemoglobin
HDL cholesterol: high-density lipoprotein cholesterol
LDL cholesterol: low-density lipoprotein cholesterol
HI: high-intensity
HOMA-IR: homeostatic model assessment of insulin resistance
HP: high protein
LED: low-energy diet
MI: moderate intensity
MP: moderate protein
OGTT: oral glucose tolerance test
PA: physical activity
SOP: Standard Operation Procedure
SBP: systolic blood pressure
DBP: diastolic blood pressure
T2D: Type 2 diabetes mellitus
TG: Triglyceride
TyG: Triglyceride glucose index
TyG-Waist: Triglyceride waist circumference index.
