# Supplementary figures and images for: Appraisal of Triglyceride-Related Markers as Early Predictors of Metabolic Outcomes in the PREVIEW Lifestyle Intervention: A Controlled Post-hoc Trial
Source: Front Nutr. 2021 Nov 1;8:733697. doi: 10.3389/fnut.2021.733697 (PMC8592084; doi:10.3389/fnut.2021.733697)

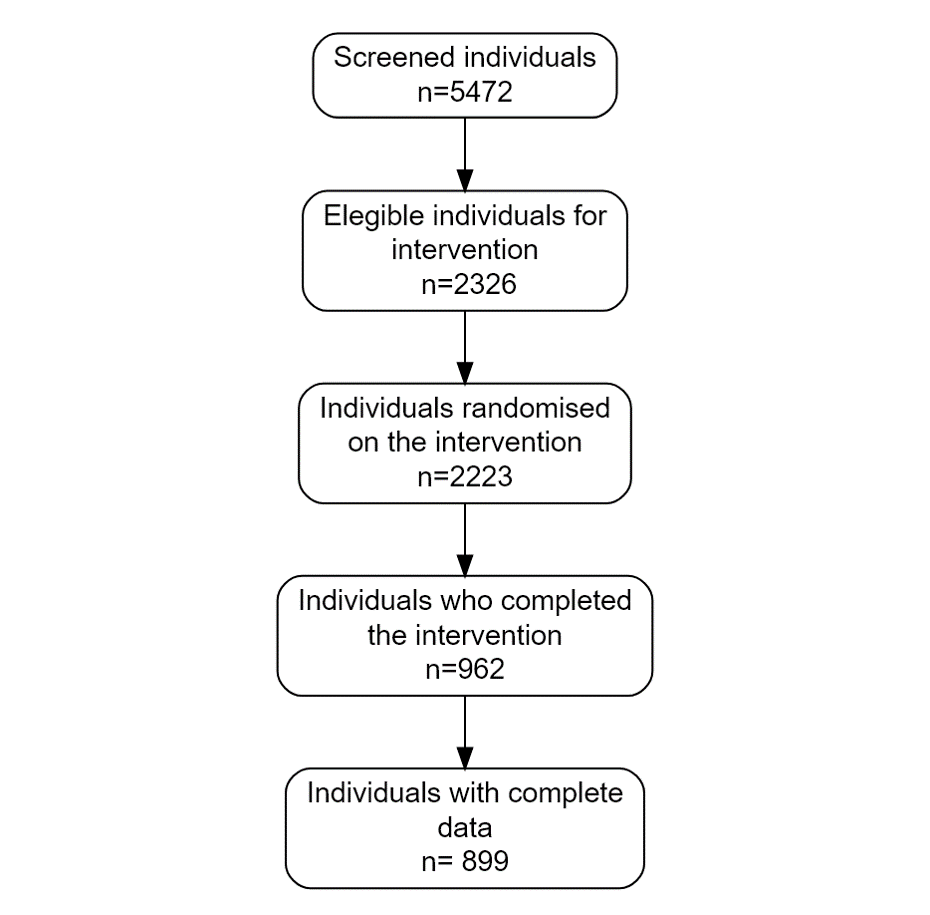

Supplement: Supplementary Figure 1 — Flowchart of the volunteers involved in the study, and whose data were analysed in the present article (Screened, Eligible and Completers, as well as those with valid full data). [file Image_1.PNG]
